# Cervical cancer screening, treatment and prophylaxis in Brazil: Current and future perspectives for cervical cancer elimination

**DOI:** 10.3389/fmed.2022.945621

**Published:** 2022-08-24

**Authors:** Flávia M. Corrêa, Arn Migowski, Liz M. de Almeida, Marcelo A. Soares

**Affiliations:** ^1^Cancer Early Detection Division, Brazilian National Cancer Institute (INCA), Rio de Janeiro, Brazil; ^2^Coordination of Prevention and Surveillance, Brazilian National Cancer Institute (INCA), Rio de Janeiro, Brazil; ^3^Oncovirology Program, Brazilian National Cancer Institute (INCA), Rio de Janeiro, Brazil; ^4^Department of Genetics, Universidade Federal Rio de Janeiro (UFRJ), Rio de Janeiro, Brazil

**Keywords:** cervical cancer, LMICs, Brazil, HPV, HPV vaccine

## Abstract

As a middle-income country, Brazil has one of the largest public health systems worldwide, which deals with free and universal access to health care. Regarding cervical cancer, the country possesses a large infrastructure for the screening of premalignant and malignant lesions, but yet based on old technology, having Papanicolaou as the major screening method, followed by colposcopy and treatment. Also, large disparities in access are present, which makes effectiveness of screening and treatment in different regions of the country highly unequal. In this review, we describe and evaluate the current screening, treatment and prophylactic (HPV vaccination) strategies to combat cervical cancer in Brazil, and discuss potential incorporation of more recent technologies in these areas in the country to pave its way toward cervical cancer elimination.

## Introduction

The history of actions for cervical cancer (CC) control in Brazil begins in the 1940's, with the introduction of colposcopy and cytology. During the following decade, those methods were disseminated throughout the country, albeit restricted to opportunistic assessment of women visiting health services for other reasons. In the 1960's, the first CC detection campaigns using the Papanicolaou test were launched, expanding in the following decades (1).

Public policies aimed at CC control were just developed from the second half of the 1970's onward, when the disease was finally recognized as a public health problem. In the 1980's, during the country's return to democracy, the healthcare reform and the growing force of women's movements enabled the Comprehensive Women's Health Program development by the Brazilian Ministry of Health (BMoH). In 1995, the BMoH acknowledged the need of a nationwide program targeting CC control. Thus, the *Viva Mulher* (Live Woman) Program was created: the pilot project, the Cervical Cancer Information System (SISCOLO) implementation, and the program intensification phases one and two took place in 1996, 1998, 1999, and 2002, respectively. In 2005/2006, with the National Oncology Care Policy and the Health Pact launched, CC control also became part of state and municipal healthcare plans, encompassing the three government domains (1, 2).

Considering CC relevance persistence in Brazil, the BMoH elaborated and implemented a national plan to strengthen the prevention, diagnosis, and treatment network between 2010 and 2014. That included several actions such as the publication of the new National Cancer Prevention and Control Policy and the Brazilian Guidelines for the Cervical Cancer Screening update; the launch of the Cancer Information System (SISCAN) new web-based version dedicated to national screening programs; the redefinition of National Qualification in Cytopathology standards; the implementation of the Reference Services for Cervical Cancer Precursor Lesions Diagnosis and Treatment; and the human papillomavirus (HPV) vaccine incorporation into the National Immunization Program (PNI in the Portuguese acronym) (2).

Although all these efforts, cervical cancer still represents a major public health concern in Brazil. It is the third most common cancer in women and the fourth leading cancer death cause (3). In 2022, 16,710 new cases are estimated, with a rate of 16.35 cases/100,000 women (3). In 2020, there were 6,627 deaths from CC in the country, with a mortality rate of 5.33/100,000 women (4). In 2019, 160.8 disability-adjusted life-years (DALYs)/100,000 inhabitants were lost due to CC (5).

Despite the availability of HPV vaccination and CC screening free of charge in the Public Health System (SUS in the Portuguese acronym), the impact on disease magnitude has been minor, as vaccination coverage is low (6) and its effect on incidence and mortality occurs only in the long run. Consequently, screening remains an essential strategy as unvaccinated cohorts have a higher risk of developing CC and rely exclusively on early detection. However, screening remains opportunistic and cytology-based in Brazil, with challenges yet unmet to improve adherence and quality (7).

In 2018, the World Health Organization (WHO) made a global call to eliminate CC as a public health problem, and in 2020 it launched strategies to promote and accelerate this purpose (8, 9). One of the actions listed was the WHO Guideline for Screening and Treatment of Cervical Pre-cancer Lesions for Cervical Cancer Prevention review. The update process was based on Health Technology Assessment (HTA) and built from systematic literature reviews and cost-effectiveness analyses, focused on evaluating the benefits and harms associated with different alternatives, and conducted with extreme methodological rigor. The second edition of the guideline was published in July, 2021 (10).

Although CC elimination is a WHO priority, the impact of the new coronavirus disease (SARS-CoV-2/covid-19) pandemic may compromise the achievement of the proposed goals, as elective care was momentarily interrupted, causing a backlog (11). In Brazil, almost all cancer screening, diagnostic investigation and treatment related procedures decreased in 2020 compared to those recorded in 2019. Cervical cytopathological exams dropped 42% (12). However, the pandemic can be considered an opportunity to reassess CC control actions, increasing HPV vaccination coverage, promote screening organization and incorporation of novel technologies (13).

**Figure 1:**
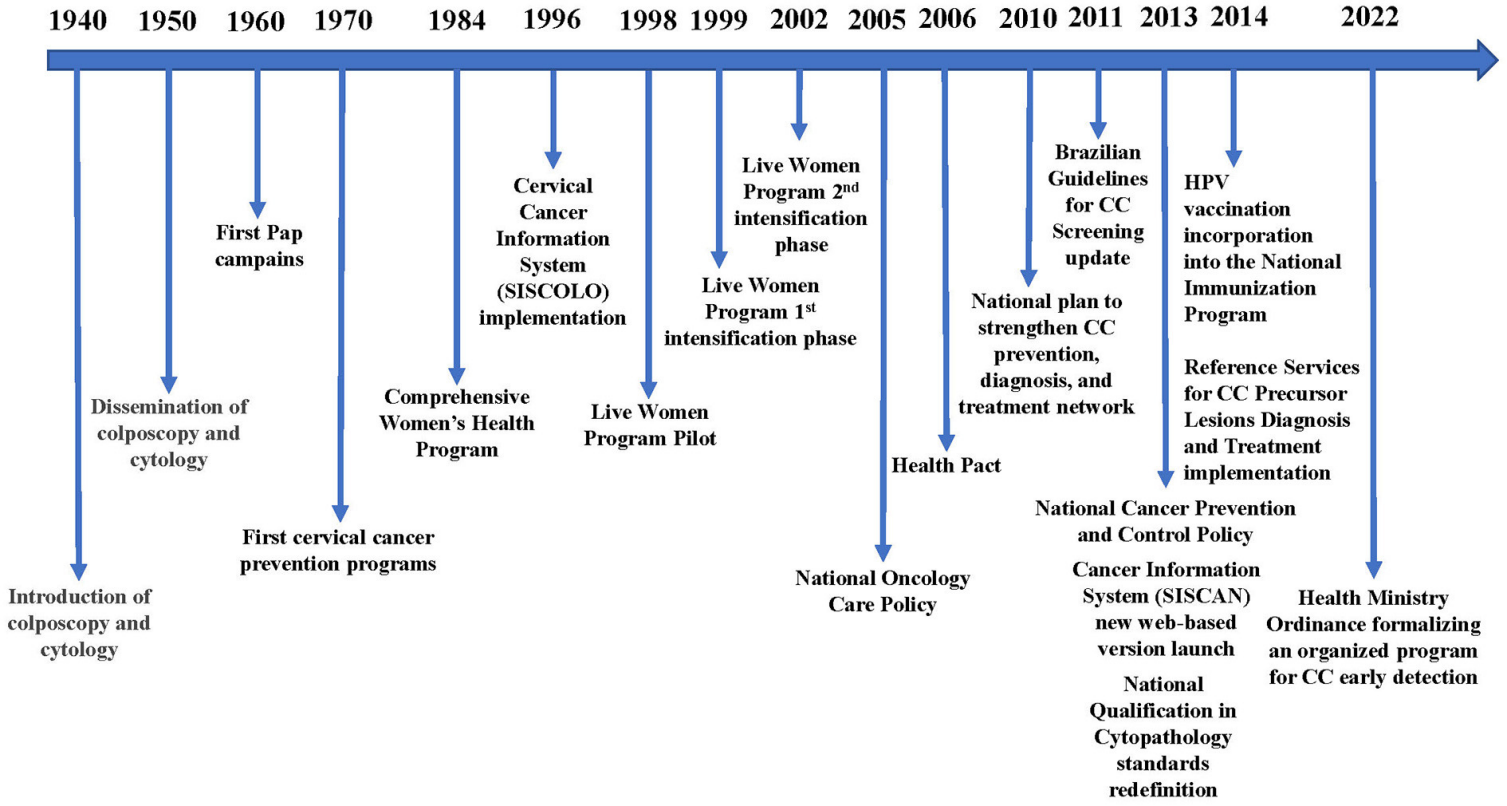
Timeline of cervical cancer prevention actions in Brazil.

A timeline of cervical cancer prevention actions in Brazil is summarized in Figure 1.

## Cervical cancer mitigation through vaccination, screening, and treatment

### Prophylactic HPV vaccination in Brazil

HPV vaccines have been approved for distribution in Brazil through the SUS in late 2013. By March 2014, the first doses were distributed free of charge for young girls aged 11–13 yrs-old. One year later (2015), girls aged 9–10 yrs-old were also included in the PNI. Currently, the BMoH offers vaccination to girls aged 9–14 yrs-old (14). Women living with HIV (WLWH), solid-organ and bone marrow transplant recipients and cancer patients aged 9–45 yrs-old are also offered HPV vaccination (15). Since 2017, the distribution of the vaccine for young boys has also been approved, firstly for the 12–13 yrs-old range, and then expanded from 11 to 14 yrs-old (14). Likewise, young men living with HIV or under immunosuppression conditions from 9 to 26 yrs-old are also eligible for HPV vaccination (14).

Two vaccines have been initially approved in the country, the quadrivalent (qHPV, Gardasil^®^), protecting against HPV types 6, 11, 16, and 18; and the bivalent (bHPV, Cervarix^®^), protecting only against the high-risk oncogenic HPV types 16 and 18. These vaccines have been approved by the FDA in 2006 and 2009, respectively, denoting a significant delay in implementing HPV vaccination in large scale in Brazil. The BMoH adopted mostly the quadrivalent HPV vaccine for distribution within SUS, as it also includes protection against the development of anogenital warts in women and men induced by the low-risk HPV types 6 and 11. For girls and boys, two doses (0 and 6 months) are recommended; for those immunosuppressed (9–45 yrs-old), three doses are prescribed (0, 2 and 6 months). A third HPV vaccine, the non-avalent one (encompassing the HPV types 6, 11, 16, 18 present in the qHPV and additionally the types 31, 33, 45, 52, and 58; nHPV, Gardasil-9^®^), has also been developed, but is not incorporated into the Brazilian SUS free of charge. The additional HPV types covered by this vaccine protect for an additional 20% of cervical cancer cases induced by infection with those HPV types, and its adoption by the Brazilian government would be desirable, pending cost-effectiveness and budget impact analyses.

Maintaining HPV vaccination coverage constitutes a major challenge for most developing countries, and Brazil is no different in that regard. When comparing the two 1st years after the introduction of the HPV vaccine in Brazil (2014 vs. 2015), a reduction of 22% in coverage of the first dose has been observed (92 vs. 70%). In the 1st year of vaccination for boys (2017), the coverage of the first dose in that group was only 44% (16). Despite the fact that men can, in principle, benefit from the herd immunity provided by vaccination of women, this concept does not apply to men who have sex with men, who are not reachable by herd immunity and must rely on good vaccination coverage among boys (17).

As a country with continental dimensions, Brazil has also been impacted by socio-demographic and spacio-geographic factors that promoted low coverage HPV vaccination. In the 1st year of vaccination (2014), 87% of the Brazilian municipalities had achieved the goal target of 80% coverage for the first dose among eligible girls; this number dropped to 32% of the cities for the second dose (18). Numerous individual factors have been described as associated with low HPV vaccine coverage, including low educational level, low income, countryside residence and low access to information and health services (19–22). At a population level, social/structural determinants are paramount, such as living conditions, presence of sewage, piped water, garbage collection, etc. Along those lines, a study conducted with data from 2014 to 2017 across three cohorts of girls under eligibility for vaccination in that period showed Brazilian micro-regions of low coverage for the first dose in the North region of the country, particularly in the state of Amazonas and in some parts of Pará state (23). Coverage for the second dose followed the same socio-economic patterns, but with generalized lower percentages across the country compared to the first dose, as expected. In summary, a great impact of the social inequality across Brazil was seen on the spatial heterogeneity of the HPV vaccine coverage, urging for specific planning of strategies for each territory by state health authorities, including the expansion for a school-based program (23, 24).

Recently, the WHO Strategic Advisory Group of Experts (SAGE) on Immunization reviewed real-world evidence on the efficacy of a single dose HPV vaccine schedule and advised that countries may adopt it for 9–14-year-old girls (25). This alternative schedule is less resource-intensive and easier to implement from the public health prospect, and could be evaluated in the Brazilian context.

### Cervical cancer screening

Strategies aimed at increasing vaccination coverage would have minimal impact on cervical cancer incidence by 2030. As the impact of vaccination is long-term, it is essential to combine this strategy with screening if the WHO targets are to be achieved. Scaling up twice-lifetime cancer screening and treatment in addition to vaccination would result in a 34.2% mortality reduction by 2030 in low- and middle-income countries (LMICs) (26). A modeling study estimated that with 20–45% vaccination coverage and 25–70% once-per-lifetime screening coverage by 2030, followed by an increase in vaccination coverage to 40–90% and in once-per-lifetime screening coverage to 90% by 2050, the average cervical cancer incidence rates would decline to 1.3 per 100,000 for high-human development index (HDI) countries like Brazil (27). According to this modeling study, assuming a minimum twice in lifetime (at 35 and 45 years) CC screening in Brazil, the target incidence rates of 4 per 100,000 would be reached between the years 2070 and 2075. However, the baseline screening coverage considered for Brazil in the model was 82% and several other relevant issues were not considered, like timely access to the diagnostic confirmation and subsequent treatment of pre-malignant lesions or cervical cancer.

Although the results of the last national pre-pandemic health survey indicated that 81.3% of Brazilian women aged between 25 and 64 years underwent cervical cytopathological examination in the last 3 years, these are self-reported values that probably reflect some degree of overestimation (28). Moreover, this coverage is heterogeneous in the country, with lower coverage in regions with higher incidence and mortality rates and even lower coverage in socioeconomically disadvantaged groups in these regions (28). Another problem is the challenge of maintaining the quality of screening throughout the national territory. Although, in general, the percentage of unsatisfactory exams or with inconclusive results is within the expected parameters, indicators such as the positivity index and the proportion of high-grade intraepithelial lesion (HSIL) among the satisfactory exams indicate that the sensitivity of the screening is still below the desired level in some regions and in some cases the positivity index is inflated by inconclusive results such as atypical squamous cells (28, 29). Thus, the real impact of effective screening strategies in these regions and among women never screened will probably be higher than predicted by the models.

HPV detection tests were recommended by WHO as the primary screening method for both the general population of women and WLWH (9), as they are more effective than cytology in reducing CC incidence and mortality, due to higher sensitivity, negative predictive value (NPV) and reproducibility (30, 31). Although HPV detection tests have not yet been incorporated into SUS, recommendations to guide professionals working in scenarios in which these tests are available have already been published, so they can be used based on scientific evidence and according to best practices (32). Additionally, studies investigating the molecular approach shift in CC screening in the Brazilian context have been conducted and published (33–37).

Incorporating molecular tests into screening would bring some interesting perspectives such as automation and increasing the age of onset from 25 to 30 years and the interval between screening tests from triennial to quinquennial, due to high NPV and high sensitivity. For this to occur, the change in technology must be linked to the change in the organizational model, with the migration from opportunistic to population-based screening. In the current model of opportunistic screening, a significant lack of adherence to target population and screening interval recommendations persists. In the SUS, around 20% of screening cytopathological exams are performed outside the target population and 73% are performed outside the recommended interval, usually annually (29, 38). This inadequacy results in a screening model less efficient than it should be, even with the current technology. Several barriers to the implementation of the current guidelines for cervical cancer screening in Brazil, as seen from the public health manager perspective, have already been identified recently, of which the main ones were the low adherence of health professionals to the guidelines in the context of the opportunistic screening and the disorganization of the health services (39). Switching to molecular methods without organizational change would remove some of the key benefits of the new technology and could exacerbate overscreening.

An interesting perspective of the new screening methods is the possibility of incorporating self-sampling, which can reduce geographic access barriers, minimize cultural resistance to being examined by a health professional and favor the active search of women who do not regularly attend health services. Several strategies for implementing self-sampling are being studied (40), but their effectiveness may be impacted by local issues like educational level, health literacy and other cultural and socioeconomic issues.

SUS primary care covers 76% of the country's population (41). The Family Health Strategy is organized by geographic regions and composed by multi-professional healthcare teams, including community health workers. There is evidence on the impact of this strategy on mortality of adults aged 25–64 years (42). Family health teams register the population assigned to their territory and community health workers carry out periodic home visits, which could be used for active individual invitations to population-based screening.

Another improvement needed is the integration of SISCAN with the primary care information system (Sisab). This linkage between systems will allow the identification of the target-population and the organization of individual invitations for screening in primary care settings throughout the country. Moreover, the new mobile application of the Ministry of Health, called “ConecteSUS,” allows the direct interaction of health services with women and could also be used to invite the target-population for screening at the correct intervals and also for recall in case of positive exams.

Remuneration per procedure is the logic still in force in the country, with publicly funded screening even when performed outside the target population or recommended interval. To induce a shift to an organized screening program, this funding model should be modified to be based on the needs of the entire line of care, involving screening, diagnostic confirmation and treatment. In this new financing model, coverage of the target population, the follow-up of women with positive screening and the achievement of quality indicator targets would be encouraged.

In Brazil, there are some pilot experiences with new screening methods and better organization of screening (33–37, 43). Since 2012 an organized CC screening has been established in the interior of the State of São Paulo (18 municipalities in Barreto's region) (43). A computerized system enables the institutions to send letters inviting all women within the target-population to liquid-based cytology screening. The system identifies each woman's last cytology test and automatically generates the date on which she must repeat the exam. Women receive the cytology result and the next appointment date at their home address. Women with abnormal screening tests requiring recall for further assessment are automatically listed and have the appointment scheduled. Additionally, the system identifies women who are not up to date with their screening tests, generating convocation letters. The system also records sociodemographic information; previously performed cervical cytology screening results; quality indicators for monitoring both sample collection and laboratory analysis; follow-up and additional tests, such as colposcopy and biopsies and respective results. The collected exams percentage increased from 54.6% in 2012 to 62.4% in 2013, to 68.4% in 2014, and to 71% in 2015. Only 5% of all carcinomas were detected at stages III/IV and 98% of women with abnormal results attended colposcopy. However, screening coverage has not reached the 70% target.

Another recent initiative is the “PREVENTIVO” program (PREvention of HPV Viruses in ENTire Indaiatuba by Vaccination and Organization of Screening), a pivotal demonstration study developed in a real-life scenario in a Brazilian city, comparing CC population-based screening using primary DNA-HPV testing with the traditional opportunistic cytology-based strategy in the public health system context (35). The program achieved high coverage (80%) and age compliance (99.2%, compared to 78% for cytology). The HPV-based screening detected 21 women with CC with a mean age of 39.6 years, and 67% of cancers were early-stage compared to 12 CC cases detected by cytology (*p* = 0.0284) with a mean age of 49.3 years (*p* = 0.0158), and one case of early-stage (*p* = 0.0014). Furthermore, Indaiatuba's new program has proven more cost-effective than the conventional cytology-based screening (44).

The country has 5,570 municipalities and the great challenge is how to scale up organized screening programs. An innovative approach could be to link the use of new technologies to the change in the organization model and national and state managers to enable municipal managers to adhere to the new model based on eligibility criteria. A new ordinance by the Brazilian Ministry of Health, which formalized an organized program for the early detection of cervical cancer, could be an initial step toward this new model (45), although a general environment of encouraging the performance of screening tests outside the target population persists.

Other major barriers to the effectiveness of screening in Brazil are the access bottlenecks to diagnostic confirmation and treatment of precursor lesions. National pre-pandemic SUS data showed a deficit of 7.3% for colposcopy, 20.4% for biopsies, and 74.8% for type 1 and 2 excision of the transformation zone (ETZ) on an outpatient basis and 67.6% for type 2 ETZ and type 3 ETZ in hospitals (46). A recent study, carried out in a municipality with a high incidence of cervical cancer in the North of the country, showed 27.1% without evidence of any follow-up and 74.3% without complete work-up 10 months after screening among women with HSIL+ screening results (47). The entire process is very fragmented, and a woman who needs diagnostic work-up and treatment will generally require nine visits to health services, with an average time between cytology screening and colposcopy of 105 days, 40 days between biopsy and the histopathological report and additional 150 days until a high-grade lesion treatment (48). Therefore, the change in technology should also be considered in conjunction with protocols that reduce the number of steps in this process, in order to increase adherence. Furthermore, the switch to screening by HPV tests needs to be carried out in an organized program and complemented by a triage test because HPV infection is commonly found, so that there is no risk of aggravating the bottleneck in accessing diagnostic confirmation.

WHO guideline suggests triage of HPV DNA-positive women using cytology, partial genotyping, colposcopy or visual inspection with acetic acid (VIA). As the benefits, harms and programmatic costs of these triage methods are similar, the choice depends on feasibility, training, program quality assurance and resources (9). From the Brazilian perspective a recent review suggests that reflex liquid-based cytology or partial genotyping should be performed after a positive HPV test to avoid unnecessary colposcopies and follow-up losses (32). Other triage options under investigation are mRNA-HPV, extended genotyping, dual staining, and methylation (49).

Even if effective and efficient triage of HPV DNA-positive women is available, in certain settings of the country colposcopy and biopsy will remain a burden, and a screen-and-treat approach could be considered (9). Furthermore, new promising optical techniques/imaging methods are in development, such as low cost portable digital colposcopes, high resolution microendoscopes, and the use of smartphone technology and artificial intelligence/deep learning algorithms for automated interpretation of cervical images (50, 51).

### Treatment

#### Therapeutic HPV vaccination and the Brazilian perspective

All currently approved prophylactic HPV vaccines induce anti-HPV immunity through recombinant viral capsid proteins, L1 and L2 (52, 53). However, these viral proteins are not or are very rarely expressed in cells transformed with HPV. Therefore, subjects with persistent oncogenic HPV infection or HPV-associated established cancers do not benefit from vaccination with the current products (54, 55).

Therapeutic HPV vaccines should be based on viral products that are expressed in malignant cells, such as the viral oncogenic proteins E6 and E7, the major drivers of HPV-mediated oncogenesis (56, 57). Yet no product has been yet approved for clinical use anywhere in the world, research on therapeutic HPV vaccines are increasing over time, and over a 100 initiatives have already been registered at ClinicalTrials.gov (58), being most of them DNA vaccine products encoding the abovementioned viral oncoproteins or capsid proteins and using different delivery strategies. DNA vaccines are able to induce cytotoxic T-lymphocyte (CTL) responses that specifically target epitopes of these proteins expressed in HPV-transformed cells, leading to their elimination (59).

So far, two DNA vaccine candidates against HSIL have reached phase III clinical trials in Mexico and U.S. One of them, MVA E2, has been designed to induce cross-protective immunity to HPV using bovine papillomavirus (BHV)-specific E2 antigen inserted into a modified vaccinia Ankara (MVA) virus. The largest phase III trial enrolled over 1,300 subjects (mostly women) who received six injections of the immunogen in the malignant tissue. Almost 90% of the vaccinated women eliminated their lesions, and 80% cleared the oncogenic HPV associated with the disease (60). The other vaccine candidate (VGX-3100), using a completely distinct strategy, is based on naked plasmid DNA molecules carrying the genes that encode E6 and E7 from high-risk HPV types 16 and 18 and a delivery system using electroporation (61). In a modified intent-to-treat analysis with 193 patients recently publicized (62), 23.7% of patients in the vaccinee group responded with HSIL regression and HPV clearance at week 36, compared to only 11.3% of patients in the placebo group. Noteworthy, this vaccine has already shown efficacy against anal and vulvar displasia associated with HPV infection during phase II trials (63, 64).

Brazil has also developed anti-HPV therapeutic vaccine candidates in the latest decade. The most promising candidate has just been publicized by researchers from the University of São Paulo and other centers (65). The use of a recombinant fusion protein between HPV16 E7 and the herpes simplex virus 1 glycoprotein D (gD), either in the form of purified protein or recombinant DNA encoding this fusion, together with cisplatin treatment (the standard-of-care treatment for cervical cancer stages IB through IV) resulted in a synergistic control of HPV-associated advanced-stage tumors with reduced toxic effects in C57BL/6 mice. Moreover, the combined use of cisplatin and gDE7 protein induced tumor infiltration of immunomodulatory cell subsets and E7-specific CD8+ T-cells, and reduced the frequency of myeloid suppressor cells. Finally, such combination also induced synergistic therapeutic effects in advanced tumors as well as immunological memory responses and long-term protection from tumor relapses at different anatomical sites (65). The “chemo-vaccination” strategy proposed by the authors is a promising approach for the treatment of cervical cancer and likely to be further evaluated in early-phase clinical trials.

#### Precancerous lesions

CC precursor lesions treatment options include ablation with cryotherapy or thermal ablation, and excisional methods as loop electrosurgical excision procedure (LEEP) or cold knife conization (CKC). Meta-analyses comparing the efficacy of these treatments showed similar performance (66, 67). Consequently, the method of choice should be based on available resources.

Ablative therapy is cheaper, safer, and simpler to use, can be performed by non-medical providers without anesthesia, but requires cervical visual assessment because it is recommended just if the cervical lesion is entirely visible, does not extend to the endocervix or the vaginal wall, and is totally covered by the probe tip. Cryotherapy relies on gas (carbon dioxide or nitrous oxygen) supply, which is difficult to procure and transport, although portable devices with built-in gas systems have already been developed. Thermal ablation is recommended by WHO in LMICs because it is technically easier to deliver than cryotherapy as the devices can be battery-operated, are lightweight, and have a shorter treatment time. Ablation is ideal for point of care treatments at the primary care level or for a screen-and-treat approach, and could be considered in some Brazilian settings (50, 51).

Excision is recommended if there is a contraindication for ablation, and is often preferred in high-resource settings because it allows histopathologic diagnosis. However, adverse events such as bleeding, infection, and obstetric complications (preterm delivery and perinatal mortality) are more frequent. Additionally, LEEP and CKC require medical training, anesthesia, hemostatic agents, electrical supply, diathermy machine, and loop electrodes (50, 51).

#### Cervical cancer

The advances in oncological care in the SUS in recent decades are undeniable (68). However, barriers to timely access to treatment still persist. In 2019, 48.3% of women had more than 60 days between the diagnosis of cervical cancer and the start of treatment in the SUS and 22.8% had no information on treatment (69). During the pandemic, there was, paradoxically, some improvement with a drop in this percentage to 45.7% treated after 60 days (69), which may be related to the lower demand due reduction of diagnosed cases and less treatments (12).

A prospective cohort study of newly diagnosed patients with CC recruited at 16 Brazilian sites representing the five Brazilian regions found that most patients were diagnosed with locally advanced or metastatic disease (FIGO clinical stage II-IV in 81.8%, stage II in 35.2%, stage III in 36.1%, and stage IV in 10.5%) (70).

Early stage cancers (FIGO stages IA1, IA2, IB1), and in some selected cases stages IB2 and IIA1 may be treated by surgery alone or by a combination of surgery and adjuvant therapy. Locally advanced disease (FIGO IB2-IVA) requires chemoradiation plus high-dose-rate (HDR) brachytherapy. For metastatic and recurrent disease angiogenesis blockade and immunotherapy with checkpoint inhibitors and other agents are currently available in high resource settings. First-line treatment for patients with recurrent and/or metastatic CC includes the association of bevacizumab with chemotherapy. Important advances have been demonstrated in the last decade for second-line treatment with immunotherapy (71–73).

Notwithstanding, in 2016 the Brazilian health technology assessment (HTA) agency (Conitec in the Portuguese acronym) recommended against incorporating bevacizumab for the treatment of persistent, recurrent or metastatic CC in SUS, as the treatment was not considered cost-effective (74). Immunotherapy is not available as well. And aggravating this bottleneck regarding advanced disease, there is a shortfall in public radiotherapy services for cancer treatment in Brazil (75).

Table 1 summarizes the existing barriers to CC control in the country and lists pertaining approaches.

**Table 1:** Existing barriers in cervical cancer control in Brazil and pertaining approaches.

**Key steps**	**Barriers**	**Approaches**	**Action needed**
HPV vaccination	Low coverage	Single dose	National Immunization Program, Primary health care, and Ministry of Education articulation
		School-based program	
		Non-adherent target-population active recruitment	
		Target-population, parents, and teachers IEC	KAP studies
		Health professionals training	
	New technology availability	nHPV incorporation	CEA, BIA
Screening	Opportunistic	Populational-based organized screening	Primary and Specialized health care articulation, including information systems
		Call and recall	
		Quality assurance	
		Precursor lesions timely follow-up and treatment	
	New technologies availability	DNA-HPV, mRNA-HPV, HPV genotyping, self-sampling, dual staining, methylation, portable devices and automated visual evaluation, thermal ablation incorporation	Regulatory approval, CEA, BIA, guidelines update
	Heterogeneous settings	Resource-oriented	Guidelines update with implementation of screen and treat protocols for some clinical settings
Diagnosis	Timely access	Improvement of the integration of different health services; active follow-up of women with positive screening; decrease in the number of consultations and unnecessary visits by women to health services	Release of a new module for follow-up in SISCAN (in progress); improve the implementation of clinical regulation; guidelines update
Treatment	Timely access	General population IEC and health professional training on early diagnosis;	KAP studies;
		Reduce shortfall in oncology services	Specialized health care capacity building
	New technologies availability	Anti-HPV therapeutic vaccine development	R&D
		Target therapy and immunotherapy	Regulatory approval, CEA, BIA, guidelines update

IEC, information, education, and communication; KAP, knowledge, attitudes, and practices; nHPV, human papillomavirus ninevalent vaccine; CEA, cost-effectiveness analysis; BIA, budget impact analysis; SISCAN, Cancer Information System; R&D, research and development.

## Conclusion

The effort to eliminate CC as a public health problem in Brazil requires a combination of multiple steps and strategies regarding primary prevention, screening, diagnosis, and treatment. The incorporation of novel technologies and approaches in those fronts is expected to help driving the country toward an elimination target. New approaches include single dose HPV vaccination—recently preconized by the WHO –; the adoption of the non-avalent vaccine, accounting for an additional 20% of the CC cases; an organized screening based on a populational basis, with assured quality, timely follow-up and treatment; novel technologies of screening and triage (DNA-HPV, mRNA-HPV, HPV genotyping, dual staining, methylation); and novel methods diagnosis and treatment of precancerous lesions (pocket colposcope, automated visual evaluation, and thermal ablation). Nevertheless, the incorporation of new technologies is not enough to impact cervical cancer incidence and mortality in a continental country with heterogeneous settings ravaged by profound inequities. Programmatic changes and resource-oriented approaches at national, regional and local levels are paramount to honor the commitment made with the WHO global call.

## Author contributions

All authors collected and analyzed the regulatory, guideline, experimental data and information, prepared the first draft of the manuscript, contributed to manuscript revision, read, and approved the submitted version.

## Conflict of interest

The authors declare that the research was conducted in the absence of any commercial or financial relationships that could be construed as a potential conflict of interest.

## Publisher's note

All claims expressed in this article are solely those of the authors and do not necessarily represent those of their affiliated organizations, or those of the publisher, the editors and the reviewers. Any product that may be evaluated in this article, or claim that may be made by its manufacturer, is not guaranteed or endorsed by the publisher.
